# Alterations in Galanin-Like Immunoreactivity in the Enteric Nervous System of the Porcine Stomach Following Acrylamide Supplementation

**DOI:** 10.3390/ijms20133345

**Published:** 2019-07-08

**Authors:** Katarzyna Palus, Krystyna Makowska, Jarosław Całka

**Affiliations:** Department of Clinical Physiology, Faculty of Veterinary Medicine, University of Warmia and Mazury in Olsztyn, Olsztyn, Poland, Oczapowskiego Str. 13, 10-718 Olsztyn, Poland

**Keywords:** acrylamide, enteric nervous system, galanin, pig, stomach

## Abstract

In recent years, a significant increase in the consumption of products containing large amounts of acrylamide (e.g., chips, fries, coffee), especially among young people has been noted. The present study was created to establish the impact of acrylamide supplementation, in tolerable daily intake (TDI) dose and a dose ten times higher than TDI, on the population of galanin-like immunoreactive (GAL-LI) stomach neurons in pigs. Additionally, in the present study, the possible functional co-operation of GAL with other neuroactive substances and their role in acrylamide intoxication was investigated. Using double-labelling immunohistochemistry, alterations in the expression of GAL were examined in the porcine stomach enteric neurons after low and high doses of acrylamide supplementation. Generally, upregulation in GAL-LI immunoreactivity in both myenteric and submucous plexuses was noted in all stomach fragments studied. Additionally, the proportion of GAL-expressing cell bodies simultaneously immunoreactive to vasoactive intestinal peptide (VIP), neuronal nitric oxide synthase (nNOS) and cocaine- and amphetamine- regulated transcript peptide (CART) also increased. The results suggest neurotrophic or/and neuroprotective properties of GAL and possible co-operation of GAL with VIP, nNOS, CART in the recovery processes in the stomach enteric nervous system (ENS) neurons following acrylamide intoxication.

## 1. Introduction

Galanin (GAL) is a 29 (or 30 in humans) amino acid peptide which has widespread distribution in the central and peripheral nervous systems, as well as in peripheral tissues of numerous species, including humans [1,2,3,4]. The occurrence of GAL was observed in both nerve cell bodies and nerve fibres located in different fragments of the gastrointestinal (GI) tracts of many species [2,5,6,7]. To date, three G-protein-coupled receptors known as galanin receptors 1, 2 and 3 (GAL-R1, GAL-R2 and GAL-R3) have been described in studies which examined tissues and organs [3]. GAL participates in many physiological functions in the GI tract, such as regulation of motility, hydrochloric acid secretion, exocrine function of the pancreas and intestinal absorption [1]. GAL may act in both an inhibitory or an excitatory role depending on the fragment of GI tract, the species and the experimental conditions [8]. Moreover, previous studies have indicated that GAL as a neuroprotective factor mediates survival or regeneration after neural injury and exerts anti-inflammatory activities [9,10]. Indeed, upregulation of GAL expression in neural structures of the GI tract was demonstrated during experimentally-induced and naturally occurring intestinal inflammation [2,11]. An enhanced percentage of GAL-like (GAL-LI) immunoreactive enteric nervous system (ENS) neurons was also observed in injuries of the digestive tract as well as in toxaemia [2,12].

One of the toxins occurring in food products is acrylamide (ACM). Acrylamide is formed at high temperature in a process known as the Maillard reaction. A high content of acrylamide has been found in many food products, such as biscuits, chips, coffee and cornflakes [13]. The literature in the field contains many papers dealing with the influence of acrylamide on living organisms. Namely, acrylamide-induced axonal damage and impaired retrograde transport resulting in symptoms of neuropathy were noted in both the central and peripheral nervous systems. [14]. Furthermore, acrylamide contributes to the creation of oxidative stress condition [15]. It is assumed that oxidative stress and an insufficient amount of antioxidants are responsible for the gastric mucosal damage [16]. The GI tract, the principal place of absorption of acrylamide, is thus directly exposed to the irritant effects of acrylamide, as well as systemic toxicity of its metabolite glycidamide [17]. According to the World Health Organization (WHO) data, the daily intake of acrylamide contained in food products ranges between 0.3 to 0.8 μg/kg of body weight [18]. It should be emphasized that the choice of the pig as a model animal, as well as young animals in the present experiment, was not accidental. The pig is an omnivorous animal, and the structure of the gastrointestinal tract and physiological processes occurring in it are similar to humans, which makes them a good animal model in biomedical research, especially concerning GI pathology [19,20]. In turn, products with a high content of acrylamide (like chips) are very popular, especially among young consumers, which makes them particularly vulnerable to their toxic effects.

On the other hand, it is known that both extrinsic (sympathetic, parasympathetic and sensory) as well as intrinsic (localized in the GI wall and forming the enteric nervous system (ENS)) neurons participate in the neural regulation of the digestive tract function. Based on autonomy, the huge number of neurons that create enteric plexuses, as well as numerous neurotransmitters synthesized and released by neurons, the ENS has been called the intestinal brain [21]. Moreover, the ENS is one of the first barriers of the organism against toxins in food. The ENS anatomy mainly depends on the animal species, as well as the part of the GI tract. In pigs and other large animals, in contrast to rodents, in the oesophagus and stomach, the ENS is arranged into two plexuses: The myenteric plexus (MP) and the submucous plexus (SP). While in the intestines, the SP is divided into two submucosal plexuses: The outer submucous plexus (OSP) and inner submucous plexus (ISP) [12,20]. Additionally, the ENS neurons show many adaptive changes, both functional and morphological, in response to inflammatory factors, injuries or toxins referred to as neural plasticity. One of them is alteration in the immunohistochemical phenotype of ENS neurons expressed as up- or down-regulation in the expression of neuroactive substances [2]. Although changes in the neurochemical profile of ENS neurons have been reported in many diseases and gastrointestinal toxicities, there is no data on the response of ENS neurons to acrylamide supplementation, particularly GAL-expression in stomach ENS neurons subjected to acrylamide toxicity. The present study was created to establish the impact of acrylamide supplementation, in tolerable daily intake (TDI) doses and a dose ten times higher than TDI, on the population of GAL-LI stomach neurons in pigs. Additionally, the possible functional co-operation of GAL with other neuroactive substances known from their neuroprotective features (VIP, nNOS, CART) and their role in acrylamide intoxication, was also investigated in the present study.

## 2. Results

**Figure 1:**
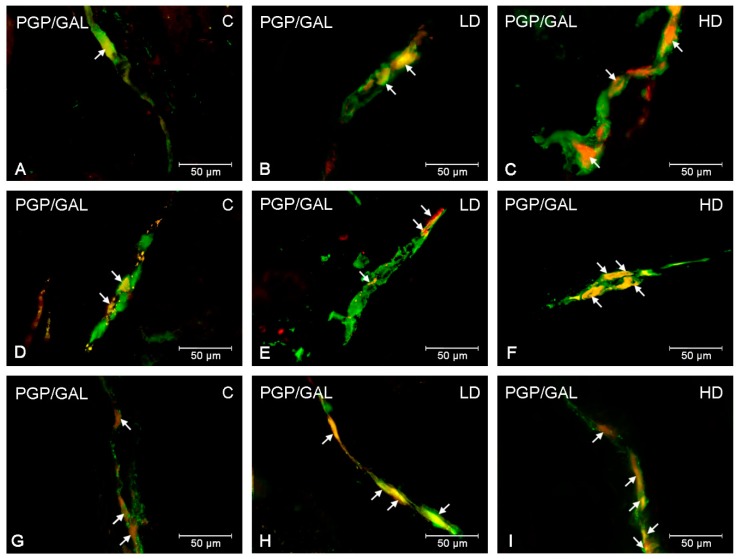
GAL-LI neurons in myenteric plexuses in the porcine stomach. The ENS neurons immunoreactive to protein gene-product 9.5 (PGP9.5)—used as a pan-neuronal marker and galanin (GAL) in myenteric plexuses in the porcine stomach under physiological condition (**A**,**D**,**G**), after low (**B**,**E**,**H**) and high doses (**C**,**F**,**I**) of acrylamide supplementation. Photographs A–C showing myenteric plexuses in the cardia, D–F—myenteric plexuses in the corpus and G–I—myenteric plexuses in the pylorus. All photographs have been created by digital superimposition of two colour channels (green for PGP 9.5 and red for GAL). Neurons immunoreactive to GAL are indicated with arrows.

**Figure 2:**
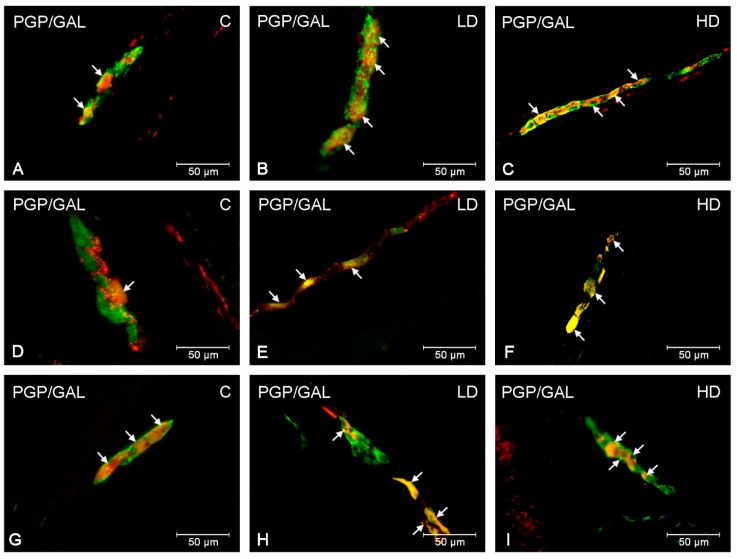
GAL-LI neurons in submucous plexuses in the porcine stomach. The ENS neurons immunoreactive to protein gene-product 9.5 (PGP9.5)—used as a pan-neuronal marker and galanin (GAL) in submucous plexuses in the porcine stomach under physiological condition (**A**,**D**,**G**), after low (**B**,**E**,**H**) and high doses (**C**,**F**,**I**) of acrylamide supplementation. Photographs A–C showing submucous plexuses in the cardia, D–F— submucous plexuses in the corpus and G–I— submucous plexuses in the pylorus. All photographs have been created by digital superimposition of two colour channels (green for PGP 9.5 and red for GAL). Neurons immunoreactive to GAL are indicated with arrows.

During the present experiment, neurons displaying immunoreactivity to GAL were observed in each part of the stomach in animals from the control and experimental groups. The occurrence of GAL-LI cell bodies was detected in both the myenteric plexus (MP) and submucous plexus (SP) in all studied stomach fragments (Table 1, Figure 1 and Figure 2). The most numerous populations of GAL-LI neurons with reference to all cells immunoreactive to protein gene product 9.5 (PGP 9.5) was found in the MP of the pylorus and cardia (25.14 ± 1.15% and 24.67 ± 0.67%, respectively) (Figure 1A,G). A slightly smaller number of GAL-positive cell bodies was noted in the corpus (19.71 ± 0.70%) (Figure 1D). In turn, in the SP, the largest group of GAL-LI nerve cells was found in the corpus (40.92 ± 0.84%), slightly smaller in the cardia (37.36 ± 0.71%), and the smallest was in the pylorus (36.87 ± 1.11%) (Figure 2A,D,G).

The supplementation of low and high doses of acrylamide affected the number of GAL-LI neurons in both the MP and SP in all studied fragments of the stomach (Table 1). The character of the observed changes depended on the type of enteric plexus and the segment of the stomach investigated. In particular, the most remarkable changes in the number of GAL-LI neurons, relative to control animals, was observed in the MP of the corpus in both experimental groups (an increase approximately 8 percentage points (pp) in the low dose (LD) and 14 pp in the high dose (HD) group, respectively) (Figure 1E,F). Similarly, in the SP of corpus, an increase in the expression of GAL was the most significant (approximately 11pp in the LD group and 18 pp in the HD group) (Figure 2E,F). A slightly smaller alteration was noted in the MP of the cardia (an increase of approximately 5 pp in the LD group and approximately 11 pp in the HD group) (Figure 1B,C) and pylorus (elevated approximately 8 pp in the LD group and 12 pp in the HD group) (Figure 1H,I). Comparable changes were observed in the gastric SP, where the increase in GAL-LI cell bodies was statistically significant (in the cardia, approximately 3 pp in LD group and 9 pp in the HD group; in the pylorus, approximately 4 in the LD group and 9 in the HD group, respectively) (Figure 2B,C,H,I).

**Figure 3:**
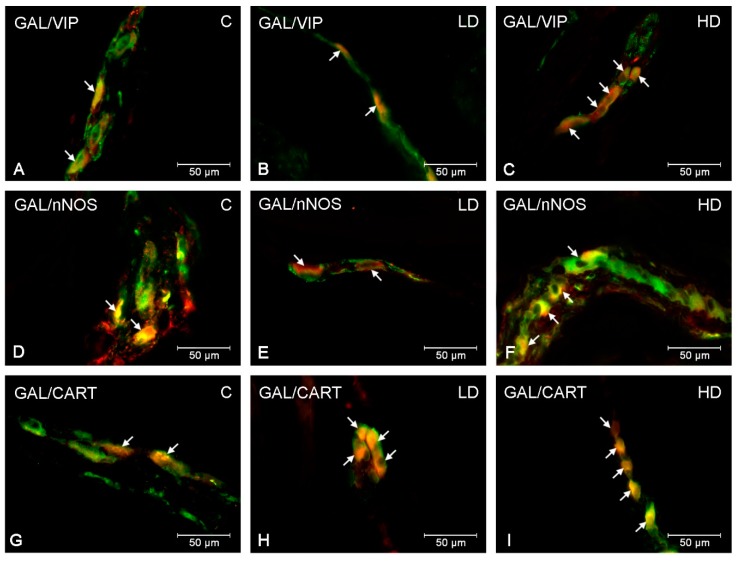
Co-localization of GAL with VIP, nNOS and CART in myenteric plexuses in the porcine stomach. The most representative images showing changes in the degree of co-localization of GAL with VIP in the myenteric plexuses of the cardia (**A**,**B**,**C**); GAL with nNOS in the myenteric plexuses of the pylorus (**D**,**E**,**F**); GAL with CART in the myenteric plexuses of the pylorus (**G**,**H**,**I**). Photographs A, D, G showing myenteric plexuses in the physiological conditions; B, E, H—after low and C, F, I after high doses of acrylamide administration. All photographs have been created by digital superimposition of two color channels (green for GAL and red for VIP, nNOS and CART respectively). Neurons immunoreactive to GAL and VIP or nNOS or CART are indicated with arrows.

**Figure 4:**
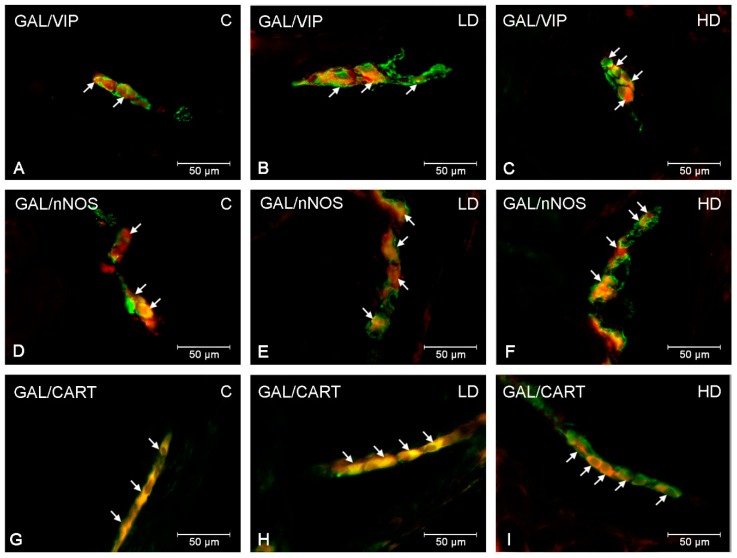
Co-localization of GAL with VIP, nNOS and CART in submucous plexuses in the porcine stomach. The most representative images showing changes in the degree of co-localization of GAL with VIP in the submucous plexuses of the corpus (**A**,**B**,**C**); GAL with nNOS in the submucous plexuses of the cardia (**D**,**E**,**F**); GAL with CART in the submucous plexuses of the cardia (**G**,**H**,**I**). Photographs A, D, G showing submucous plexuses in the physiological conditions; B, E, H—after low and C, F, I after high doses of acrylamide administration. All photographs have been created by digital superimposition of two color channels (green for GAL and red for VIP, nNOS and CART respectively). Neurons immunoreactive to GAL and VIP or nNOS or CART are indicated with arrows.

In the present study, co-localization of GAL with other neuroactive substances studied (VIP, nNOS and CART) was noted within both stomach plexuses in animals under physiological conditions, as well as after low and high doses of acrylamide supplementation. The double-labelling immunofluorescence revealed extensive co-expression of GAL with VIP in the stomach ENS neurons (Table 2). Approximately half of the GAL-LI cell bodies in the MP were simultaneously immunoreactive to VIP (46.79 ± 2.18% in the cardia (Figure 3A), 43.20 ± 1.33% in the corpus and 47.53 ± 0.67% in the pylorus). Additionally, numerous MP neurons immunoreactive to GAL also showed the presence of nNOS (33.37 ± 0.79% in the cardia, 47.77 ± 1.22% in the corpus and 32.98 ± 0.51% in the pylorus (Figure 3D)) (Table 3). The degree of co-localization of GAL with CART in MP neurons was also high and amounted to 51.68 ± 0.85% in the cardia, 32.25 ± 1.12% in the corpus and 46.29 ± 1.30 in the pylorus (Figure 1G), respectively (Table 4). In turn, in SP neurons, the highest degree of co-localization with GAL was exhibited by CART (51.34 ± 1.02% in the cardia (Figure 4G), 32.41 ± 1.36% in the corpus and 51.30 ± 1.16% in the pylorus) (Table 4). A slightly smaller number of GAL-LI perikarya was simultaneously VIP-positive (48.20 ± 0.89% in the cardia, 39.32 ± 0.69% in the corpus (Figure 4A) and 48.36 ± 1.37% in the pylorus) (Table 2). Moreover, GAL-LI neurons in the SP were also immunoreactive to nNOS in all studied stomach fragments (in the cardia 35.17 ± 0.62% (Figure 4D); in the corpus 46.16 ± 1.15; in the pylorus 37.62 ± 1.51%) (Table 3).

Acrylamide administration changed the number of GAL-LI enteric neurons simultaneously, immunoreactive to VIP, nNOS and CART in all stomach fragments studied (Table 2, Table 3 and Table 4). Both low and high doses of acrylamide substantially increased the number of SP neurons being simultaneously VIP- and GAL-positive in the stomach corpus (about 3 pp in the LD group and 10 pp in the HD group) (Figure 4B,C). However, in SP of the cardia and pylorus, significantly important alterations in VIP immunoreactivity in GAL-LI neurons were noted only in the HD group (about 12 pp in both stomach parts). In turn, in the MP, elevated numbers of GAL+/VIP+ perikarya were observed only in the HD group in all studied stomach fragments (about 14 pp in the cardia (Figure 3C), 8 pp in the corpus and 11 pp in the pylorus, respectively). In turn, nNOS-immunoreactivity in GAL-LI neurons increased the most in the pylorus and cardia where statistically relevant changes were noted in both experimental groups and stomach plexuses. In MP of the pylorus, an increase of approximately 7 pp in the LD group and 14 pp in the HD group were observed (Figure 3E,F), whereas in the cardia approximately 6 pp in the LD group and 13 pp in the HD group, respectively. In SP, in the cardia, the number of GAL+/nNOS+ neurons were elevated approximately 5 pp in the LD group and 12 pp in the HD group (Figure 4E,F), while in the pylorus the elevation was approximately 6 pp in the LD group and 10 pp in the HD group. On the other hand, in the corpus, significant changes in nNOS expression were observed in MP only in the HD group (an increase of approximately 9 pp). In turn, in the SP, similar to other parts of stomach in both LD and HD groups, the population of GAL+/nNOS+ neurons was elevated (about 4 and 11 pp, respectively). Furthermore, the expression level of CART in GAL-LI neurons was also significantly elevated. However, the character of observed changes depended on the part of the stomach as well as the kind of ENS plexus under investigation. In the MP of the cardia and corpus, only in the HD group was the increase statistically significant (approximately 15 pp in the cardia and 9 pp in the corpus), whereas changes were noticeable in the pylorus in both LD and HD group (approximately 8 pp and 10 pp, respectively) (Figure 3H,I). For SP, the most remarkable changes were noted in the cardia (an increase of approximately 8 pp in the LD group and 12 pp in the HD group) (Figure 4H,I). In the corpus, alterations in GAL+/CART+ neurons were observed only in the HD group (an increase of approximately 2 pp), although the changes observed in the pylorus were not statistically significant.

## 3. Discussion

During the present investigation, GAL- like immunoreactive neurons have been noted in both types of enteric plexuses within all stomach fragments studied. Previous studies have shown that GAL is distributed throughout the ENS of several species, including humans [2,8,11,12,22]. The occurrence of this peptide in enteric nervous structures (both neuronal cell and nerve fibers) as well as extrinsic neurons supplying the stomach was also confirmed [22,23,24,25]. The number of ENS neurons showing GAL-immunoreactivity clearly depends on the animal species and the part of the GI tract studied. Recent studies on the porcine stomach, confirmed by the results of the present experiment, have showed that GAL-positive neurons were present in a significant number of neurons in both the myenteric (MP) and submucous plexus (SP), with a slight predominance of SP [22,23]. It is well known that myenteric plexus mainly regulates the motility of the GI tract, whereas submucous plexus is responsible for the regulation of the secretion and conduction of pain reactions in the GI tract [26,27]. The above-mentioned distribution of GAL is well in line with its physiological function. In particular, GAL inhibits gastric acid and the pancreatic peptide secretions, modulates the motility of the GI tract and influences the release of other neurotransmitters [28,29]. However, the effect of its action clearly depends on both the animal species and the part of the GI tract. The inhibition of the motility in guinea pig ileum, canine pylorus and human intestines was reported [30,31,32]. In contrast, the opposite action was observed in mouse distal colon, rat stomach, as well as in the porcine and rabbit ileum [1,33,34].

Furthermore, in physiological conditions GAL-IR neurons in the ENS simultaneously showed immunoreactivity to other neurotransmitters and/or neuromodulators [35,36]. It is well known that neuroactive substances secreted in the same neurons may play similar or complementary roles [12,35]. During the present investigation, the co-localization of GAL with VIP, nNOS and CART was observed in both the SP and MP of the porcine stomach. VIP is known as a neuronal inhibitory factor within the digestive tract. It is involved in relaxation of smooth muscles, inhibits motor activity of the stomach and secretion of gastric juices but also stimulates mucus secretion, calcium ions and intestinal juice secretion [37,38]. The next substance was nNOS, an indicator of nitric oxide (NO). In the GI tract, NO inhibits smooth muscle contractility, regulates mesenteric and intestinal blood flow and participates in gut mucosal protection [39]. In turn, the physiological role of CART in the GI tract is not fully recognized. Ekblad et al. [40] suggested that CART is engaged in reducing gastric emptying, induction of stomach secretion and colonic motility. The co-localization of GAL with VIP, nNOS and CART observed in the present investigation, suggests the possible interaction of these substances in the regulation of physiological processes in the porcine stomach.

The present experiment, for the first time, has shown alterations in GAL-like immunoreactivity in the ENS neurons within the porcine stomach in response to acrylamide administration. The supplementation of TDI and ten times higher doses of acrylamide induced an increase in the number of GAL-positive neurons in both intramural plexuses (MP and SP) in all parts of the stomach under investigation. This is consistent with previous reports in which upregulated GAL- expression in the ENS structures was described in many pathological conditions within the GI tract, including inflammatory processes, tumors, neuronal damage and intoxication [2,12,22,41]. It is also in accordance with the neuronal plasticity phenomenon found in ENS neurons, defined as the ability to modify the chemical code of neurons by altering neuropeptide expression in response to changing environmental conditions, injures or other disorders [2,42].

A possible explanation of the changes observed in the present study may be the neurotoxic effects of acrylamide on the stomach ENS neurons. The results of experimental studies confirmed that acrylamide causes cell damage in the nervous and reproductive systems and leads to the occurrence of some types of cancer [43]. Particularly important, however, is its neurotoxic effects, which was examined, not only in experimental animals, but also in humans [43,44]. Until now, it has been shown that acrylamide causes swelling of distal axons leading to degeneration and, consequently, to the occurrence of peripheral neuropathy [45]. Further, acrylamide induces a neurotoxic effect by binding to cysteine-rich receptor proteins, causing nerve conduction disorders and disturbances in nerve function [43]. Although the influence of ACM on the peripheral nervous system is well known, knowledge of their impact on the ENS neurons is rather fragmentary. In particular, only our previous data have shown that even a low dose of acrylamide may induce changes in the number of enteric neurons positive to CART as well as VIP-, calcitonin gene-related peptide- and substance P–immunoreactive neuronal structures in various parts of the porcine GI tract [46,47]. It is suspected that the mechanism of neurotoxic action described in the peripheral nervous system is also similar in the case of ENS. However, this hypothesis needs to be confirmed by more detailed research. Nevertheless, the fluctuations in the number of GAL-LI neurons observed in this experiment are strongly supported by the fact that GAL is known to be an important neuroprotective factor. The association of elevated galanin expression with trophic and regenerative processes after peripheral nerve injury was described in both central and peripheral nervous systems [48]. Moreover, previous investigations have shown an increase in the number of GAL-LI structures in all intramural plexuses of the porcine descending colon under axotomy [2]. In view of the above-mentioned data, it may be speculated with a high probability that an increase in the number of GAL-LI neurons is associated with adaptive and neuroprotective changes of the enteric neurons in response to the neurotoxic effect of acrylamide. However, it is also possible that acrylamide led to damage of ENS neurons suggesting that cells lacking GAL are more vulnerable to acrylamide. In the literature, there is a lack of data describing the influence of acrylamide consumed in food products on the survival of the ENS neurons. However, an in vitro study on the cultured rats myenteric neurons did not cause death of neuronal cells but led to a decrease in number of axons [49]. It is therefore more likely that the increase in the number of GAL-LI neurons observed in the present study is the result of up-regulated synthesis of GAL in ENS neurons.

On the other hand, it is also very likely that the observed changes result from the pro-inflammatory properties of acrylamide. Chronic exposure to acrylamide in humans leads to fluctuations in the level of proinflammatory cytokines and enhanced levels of plasma C-reactive protein (CRP) [15]. Similarly, the inducible-form of nitric oxide synthase (iNOS) in the blood serum of albino mice was noted during acrylamide intoxication [50]. This corresponds well with the fact that GAL is commonly known to take part in the regulation of inflammatory processes. Accordingly, an increase in galanin expression has been noted in numerous animal models of inflammation, such as chemically-induced colitis [2] and enteric Salmonella infection [51]. Moreover, GAL modulates inflammatory responses by its effect on the secretion of cytokines, such as TNF-α, IL-1α and IL-8 [52]. Although the participation of GAL in inflammatory processes in the GI tract is unquestionable, further studies are necessary to confirm that changes observed in the course of acrylamide intoxication are associated with inflammation.

Furthermore, the present investigation, for the first time, demonstrated changes in the co-localization of GAL with other neuroactive substances in the stomach ENS neurons following acrylamide intoxication. The results showed that the proportion of GAL-expressing nerve cell bodies simultaneously immunoreactive to VIP, nNOS and CART increased. The changes were the most remarkable in the HD group. The present findings closely correlated with recent investigations in which neuroprotective properties of VIP, nNOS and CART in the ENS were demonstrated [35,40,46]. VIP is engaged in the regulation of inflammatory conditions, which closely interacts with the immune system, inhibits macrophage activity, supports Th2 cells and decreases T cell migration through intestinal Peyer’s patches [53]. As an effect of its action, an elevated level of anti-inflammatory cytokines (mainly IL-10) and reduced synthesis of pro-inflammatory factors (such as TNF α, IL-6 and IL-12) were noted [54]. Moreover, VIP promotes the survival of cultured porcine myenteric neurons [55]. Similarly, nNOS leads to an increase in the survival rate of cultured neurons from adult rat colonic submucous ganglia [56]. An increased nNOS expression has also been reported in the ENS structures during extrinsic denervation [57] as well as in inflammatory bowel disease [58]. This confirms the important role of nNOS in neuronal plasticity. Additionally, the role of CART in neuroprotection was also discussed by many authors. In some studies, upregulated CART expression in the ENS structures were reported during hypertension, inflammatory conditions or intoxication [12,47,59,60]. The neurotrophic effect of CART was also demonstrated in the CNS [40]. The results of the present experiment, together with the above-mentioned data, point towards a neuroprotective role of CART. In view of the foregoing, it is very likely that changes in GAL-positive neurons reflect neuroprotective and recovery processes in the stomach ENS neurons following acrylamide intoxication. However, the mechanisms of these changes are unknown and further study is needed to clarify this issue.

## 4. Material and Methods

### 4.1. Animals and Experimental Procedures

The experimental procedure, including animal euthanasia, was approved by the Local Committee for Animal Experiments in Olsztyn (Approval No.: 11/2017). All possible efforts were made to minimize animal suffering. The experiment was conducted on 15 sexually immature female pigs of the Danish Landrace, approximately 8 weeks old and approximately 20 kg of body weight (b.w.). The gilts were kept under regular lighting conditions in a temperature-controlled environment. They were fed by commercial grain mixture and tap water ad libitum. After a period of acclimatization, the animals were randomly assigned into three groups: Control (C group), which were given empty gelatine capsules; a low dose group (LD group), animals given capsules with a tolerable daily intake (TDI) dose of acrylamide (0.5 µg/kg b.w./day); and a high dose group (HD group), animals given capsules with acrylamide in a dose ten times higher than TDI (5 µg/kg b.w./day). The capsules in all groups of animals were administered per os, once daily before the morning feed for 28 days. In order to determine the exact dose of acrylamide per animal, on the first day of the experiment, all animals were weighed, and then weighed once a week. After a period of supplementation, all gilts were treated with azaperone (Stresnil, Jansen Pharmaceutica N.V., Belgium, 4 mg/kg of body weight, i.m.) and after 15 min euthanized using a lethal dose of sodium pentobarbital (Morbital, Biowet Puławy, Puławy, Poland; 0.6 mL/kg of body weight, i.v.). Then, fragments of the stomach, the cardia, corpus and pylorus, were collected for further analysis. The tissues were fixed in 4% buffered paraformaldehyde (pH 7.4) for 1 h, rinsed in phosphate buffer (0.1 M, pH 7.4, at 4 °C) for 3 days with the exchange of the buffer every day, and then inserted into 18% phosphate buffered sucrose (at 4 °C) for 2 weeks. The frozen samples were cut using a Microm HM 560 cryostat (Carl Zeiss, Oberkochen, Germany) into 14 μm-thick sections and mounted on gelatine-coated slides.

### 4.2. Immunofluorescence Procedure

The frozen sections were then subjected to routine double-labelling immunofluorescence (as described previously by Palus et al. [61]). The sections were briefly air-dried at room temperature for 45 min and rinsed in 0.1 M phosphate-buffered saline (PBS, pH 7.4; 3 × 10 min). Subsequently, they were incubated with buffered blocking mixture (containing 10% horse serum and 0.1% bovine serum albumin in 0.1 M PBS, 1% Triton X-100, 0.05% Thimerosal and 0.01% sodium azide) for an hour in a humid chamber at room temperature. After rinsing in PBS (3 × 10 min), the samples were incubated overnight with a combination of antisera directed towards protein gene-product 9.5 (PGP 9.5; mouse, cat. No. 7863-2004, Bio-Rad, Hercules, CA, USA, working dilution 1:1000, used here as a pan neuronal marker) and galanin (GAL, rabbit, cat. No. RIN7153, Peninsula, San Carlos, CA, USA, working dilution 1:3000), as well as GAL (guinea pig, cat. No. T-5036, Peninsula, San Carlos, CA, USA, working dilution 1:2000) and vasoactive intestinal peptide (VIP, rabbit, cat. No. 11428, Cappel, Aurora, OH, USA, working dilution 1:3000), cocaine- and amphetamine-regulated transcript peptide (CART, rabbit, cat. No. H-003-61, Phoenix Pharmaceuticals, Burlingame, CA, USA, working dilution 1:8000), neuronal nitric oxide synthase (nNOS, rabbit, cat. No. AB5380, Sigma-Aldrich, Saint Louis, MO, USA, working dilution 1:2000). On the next day, following subsequent rinsing in PBS (3 × 10 min), the sections were incubated with species-specific secondary antibodies (appropriate products: Alexa Fluor 488 (donkey anti-mouse IgG, cat. No. A21202, Invitrogen, Carlsbad, California, USA, working dilution 1:1000), Alexa Fluor 488 (donkey anti-guinea pig IgG, cat. No. A11073, Invitrogen, Carlsbad, California, USA, working dilution 1:1000) and Alexa Fluor 546 (goat anti-rabbit IgG, cat. No. A11010, Invitrogen, Carlsbad, California, USA, working dilution 1:1000) for 1 h at room temperature. Next, the washed sections (PBS, 3 × 10 min) were cover-slipped in carbonate-buffered glycerol (pH 8.6).

### 4.3. Negative Control

The standard controls, i.e., pre-absorption for the neuropeptide antisera with appropriate antigen: PGP 9.5 (AbD Serotec, Kidlington, UK), GAL (026-06, Phoenix Pharmaceutical), nNOS (N3033, Sigma, St Louis, MO, USA), VIP (064-24, Phoenix Pharmaceutical), and CART (Phoenix Pharmaceuticals, Burlingame, CA, USA) at a concentration of 20 µg/mL for 18 h at 37 °C and the omission, as well as the replacement of all primary antisera by non-immune sera, were performed to test immunohistochemical labelling. The above-mentioned controls completely eliminated labelling in the tissue.

### 4.4. Counting and Statistics

The stained preparations were then analyzed using an Olympus BX51 epi-fluorescence microscope and photographed with a digital camera connected to a PC and then processed with Olympus Cell F image-analysis software (Olympus, Tokyo, Japan). The number of the GAL-like immunoreactive (LI) enteric neurons was expressed as a percentage of the total number of PGP 9.5 positive perikarya. At least 500 PGP 9.5 labelled cell bodies in sections, at least 200 µm away from each other, were counted per animal in both the myenteric (MP) and submucosal plexus (SP) within each part of the stomach (cardia, corpus and pylorus). In order to determine the co-localization of GAL with other neuroactive substances (VIP, nNOS and CART), at least 100 GAL-positive cell bodies were investigated for immunoreactivity to particular neuronal factors. In these studies, GAL-positive neurons were considered as representing 100%. Only cells with well-visible nucleus were considered, pooled and expressed as a mean ± standard error of mean (SEM). The results from each group were analyzed statistically with Statistica 12 (StatSoft Inc., Tulsa, OK, USA). The significant differences were assessed with one-way analysis of variance (ANOVA) with Dunnett’s test (* *p* < 0.05, ** *p* < 0.01, *** *p* < 0.001).

## 5. Conclusions

In summary, the present experiment revealed that supplementation of acrylamide triggers a reaction of the porcine stomach ENS neurons, expressed as an increased number of GAL-like immunoreactive neurons. Simultaneously, an increase in the expression of VIP, nNOS and CART in GAL-LI neurons was also observed. The observed alterations may be associated with adaptive and neuroprotective changes of the enteric neurons in response to the neurotoxic effect of acrylamide or may result from the inflammatory conditions that may accompany acrylamide supplementation. This suggests neurotrophic or/and neuroprotective properties of GAL and possible co-operation of GAL with VIP, nNOS, CART in the recovery processes within the ENS. Furthermore, the marked changes observed with exposure even to a low dose (TDI) of acrylamide raise doubts about the safe dose of acrylamide for consumers. In view of the growing demand for products containing large amounts of acrylamide, especially among children, the present study may be the starting point for further toxicological, pharmacological and clinical studies to ensure consumer safety.

## Figures and Tables

**Table 1:** GAL-LI immunoreactive neurons in various parts of the porcine stomach under physiological conditions (C group) and after low dose (LD group) or high dose (HD group) of acrylamide administration.

Part of the Stomach	Cardia			Corpus			Pylorus		
	C Group	LD Group	HD Group	C Group	LD Group	HD Group	C Group	LD Group	HD Group
***MP***	24.67 ± 0.67	29.89 ± 1.08 (**)	35.35 ± 0.64 (***)	19.71 ± 0.70	27.81 ± 1.23 (***)	33.55 ± 0.92 (***)	25.14 ± 1.15	33.59 ± 1.17 (***)	37.14 ± 0.43 (***)
***SP***	37.36 ± 0.71	40.43 ± 0.70 (**)	46.92 ± 0.42 (***)	40.92 ± 0.84	51.38 ± 1.28 (***)	58.53 ± 1.63 (***)	36.87 ± 1.11	40.37 ± 0.66 (*)	45.42 ± 0.58 (***)

MP- myenteric plexus, SP- submucous plexus. Significant differences were assessed with one-way analysis of variance (ANOVA) with Dunnett’s test (* *p* < 0.05, ** *p* < 0.01, *** *p* < 0.001).

**Table 2:** Co-localization of GAL with vasoactive intestinal polypeptide (VIP) in the enteric neurons in various parts of the porcine stomach under physiological conditions (C group) and after low dose (LD group) or high dose (HD group) of acrylamide administration.

GAL/VIP									
Part of the Stomach	Cardia			Corpus			Pylorus		
	C Group	LD Group	HD Group	C Group	LD Group	HD Group	C Group	LD Group	HD Group
***MP***	46.79 ± 2.18	50.28 ± 1.55	60.67 ± 2.34 (***)	43.20 ± 1.33	45.17 ± 0.73	51.49 ± 0.89 (***)	47.53 ± 0.67	50.57 ± 0.79	58.63± 1.72 (***)
***SP***	48.20 ± 0.89	52.51 ± 1.81	60.19 ± 2.06 (***)	39.32 ± 0.69	42.34 ± 0.78 (*)	49.27 ± 0.86 (***)	48.36 ± 1.37	51.48 ± 1.24	59.95 ± 1.16 (***)

MP- myenteric plexus, SP- submucous plexus. Significant differences were assessed with one-way analysis of variance (ANOVA) with Dunnett’s test (* *p* < 0.05, ** *p* < 0.01, *** *p* < 0.001).

**Table 3:** Co-localization of GAL with nitric oxide synthase (nNOS) in the enteric neurons in various parts of the porcine stomach under physiological conditions (C group) and after low dose (LD group) or high dose (HD group) of acrylamide administration.

GAL/nNOS									
Part of the Stomach	Cardia			Corpus			Pylorus		
	C Group	LD Group	HD Group	C Group	LD Group	HD Group	C Group	LD Group	HD Group
***MP***	33.37 ± 0.79	39.28 ± 1.30 (**)	46.77 ± 1.20 (***)	47.77 ± 1.22	51.76 ± 1.46	56.81 ± 1.06 (***)	32.98 ± 0.51	39.50 ± 1.15 (**)	46.77 ± 1.38 (***)
***SP***	35.17 ± 0.62	39.92 ± 1.40 (*)	46.76 ± 0.86 (***)	46.16 ± 1.152	50.55 ± 1.23 (*)	57.23 ± 0.84 (***)	37.62 ± 1.51	42.32 ± 1.01 (*)	48.03 ± 1.14 (***)

MP- myenteric plexus, SP- submucous plexus. Significant differences were assessed with one-way analysis of variance (ANOVA) with Dunnett’s test (* *p* < 0.05, ** *p* < 0.01, *** *p* < 0.001).

**Table 4:** Co-localization of GAL with cocaine- and amphetamine-regulated transcript (CART) in the enteric neurons in various parts of the porcine stomach under physiological conditions (C group) and after low dose (LD group) or high dose (HD group) of acrylamide administration.

GAL/CART									
Part of the Stomach	Cardia			Corpus			Pylorus		
	C Group	LD Group	HD Group	C Group	LD Group	HD Group	C Group	LD Group	HD Group
***MP***	51.68 ± 0.85	54.29 ± 1.52	66.74 ± 0.48 (***)	32.25 ± 1.12	36.39 ± 1.55	40.95 ± 0.76 (***)	46.29 ± 1.30	54.60 ± 0.51 (***)	56.81± 1.65 (***)
***SP***	51.34 ± 1.02	59.06 ± 1.69 (**)	63.62 ± 0.97 (***)	32.41 ± 1.36	34.98 ± 0.67	40.59 ± 1.46 (***)	51.30 ± 1.16	51.79 ± 1.24	54.97 ± 1.23

MP- myenteric plexus, SP- submucous plexus. Significant differences were assessed with one-way analysis of variance (ANOVA) with Dunnett’s test (* *p* < 0.05, ** *p* < 0.01, *** *p* < 0.001).

## References

[1] Lang R., Gundlach A.L., Kofler B. (2007). The galanin peptide family: receptor pharmacology, pleiotropic biological actions, and implications in health and disease. Pharm. Ther..

[2] Gonkowski S., Burliński P., Skobowiat C., Majewski M., Całka J. (2010). Inflammation- and axotomy-induced changes in galanin-like immunoreactive (GAL-LI) nerve structures in the porcine descending colon. Acta Vet. Hung..

[3] Arciszewski M.B., Barabasz S., Całka J. (2008). Immunohistochemical localization of galanin receptors (GAL-R1, GAL-R2, and GAL-R3) on myenteric neurons from the sheep and dog stomach. Ann. Anat..

[4] Kisfalvi Jr I., Burghardt B., Bálint A., Zelles T., Vizi E.S., Varga G. (2000). Antisecretory effects of galanin and its putative antagonists M15, M35 and C7 in the rat stomach. J. Physiol. Paris.

[5] Tatemoto K., Rökaeus A., Jörnvall H., McDonald T.J., Mutt V. (1983). Galanin—A novel biologically active peptide from porcine intestine. Febs Lett..

[6] Hoyle C.H., Burnstock G. (1989). Galanin-like immunoreactivity in enteric neurons of the human colon. J. Anat..

[7] Ekblad E., Rökaeus A., Håkanson R., Sundler F. (1985). Galanin nerve fibers in the rat gut: distribution, origin and projections. Neuroscience.

[8] Anselmi L., Stella SLJr Lakhter A., Hirano A., Tonini M., Sternini C. (2005). Galanin receptors in the rat gastrointestinal tract. Neuropeptides.

[9] Liu H.X., Hökfelt T. (2002). The participation of galanin in pain processing at the spinal level. Trends Pharm. Sci..

[10] Locker F., Lang A.A., Koller A., Lang R., Bianchini R., Kofler B. (2015). Galanin modulates human and murine neutrophil activation in vitro. Acta Physiol..

[11] Pidsudko Z., Kaleczyc J., Wasowicz K., Sienkiewicz W., Majewski M., Zajac W., Lakomy M. (2008). Distribution and chemical coding of intramural neurons in the porcine ileum during proliferative enteropathy. J. Comp. Pathol..

[12] Szymanska K., Makowska K., Gonkowski S. (2018). The Influence of High and Low Doses of Bisphenol A (BPA) on the Enteric Nervous System of the Porcine Ileum. Int. J. Mol. Sci..

[13] Van Lancker F., Adams A., De Kimpe N. (2011). Chemical modifications of peptides and their impact on food properties. Chem. Rev..

[14] Attoff K., Kertika D., Lundqvist J., Oredsson S., Forsby A. (2016). Acrylamide affects proliferation and differentiation of the neural progenitor cell line C17.2 and the neuroblastoma cell line SH-SY5Y. Toxicol. In Vitro.

[15] Naruszewicz M., Zapolska-Downar D., Kośmider A., Nowicka G., Kozłowska-Wojciechowska M., Vikström A.S., Törnqvist M. (2009). Chronic intake of potato chips in humans increases the production of reactive oxygen radicals by leukocytes and increases plasma C-reactive protein: a pilot study. Am. J. Clin. Nutr..

[16] Bhattacharyya A., Chattopadhyay R., Mitra S., Crowe S.E. (2014). Oxidative stress: an essential factor in the pathogenesis of gastrointestinal mucosal diseases. Physiol. Rev..

[17] Zödl B., Schmid D., Wassler G., Gundacker C., Leibetseder V., Thalhammer T., Ekmekcioglu C. (2007). Intestinal transport and metabolism of acrylamide. Toxicology.

[18] WHO (2002). Health Implications of Acrylamide in Food.

[19] Verma N., Rettenmeier A.W., Schmitz-Spanke S. (2011). Recent advances in the use of Sus scrofa (pig) as a model system for proteomic studies. Proteomics.

[20] Brown D.R., Timmermans J.P. (2004). Lessons from the porcine enteric nervous system. Neurogastroenterol. Motil..

[21] Furness J.B., Callaghan B.P., Rivera L.R., Cho H.J. (2014). The enteric nervous system and gastrointestinal innervation: integrated local and central control. Adv. Exp. Med. Biol..

[22] Bulc M., Palus K., Zielonka Ł., Gajęcka M., Całka J. (2017). Changes in expression of inhibitory substances in the intramural neurons of the stomach following streptozotocin- induced diabetes in the pig. World J. Gastroenterol..

[23] Zalecki M., Sienkiewicz W., Franke-Radowiecka A., Klimczuk M., Kaleczyc J. (2016). The Influence of Gastric Antral Ulcerations on the Expression of Galanin and GalR1, GalR2, GalR3 Receptors in the Pylorus with Regard to Gastric Intrinsic Innervation of the Pyloric Sphincter. PLoS ONE..

[24] Palus K., Całka J. (2016). Neurochemical Plasticity of the Coeliac-Superior Mesenteric Ganglion Complex Neurons Projecting to the Prepyloric Area of the Porcine Stomach following Hyperacidity. Neural Plast..

[25] Gańko M., Całka J. (2014). Localization and chemical coding of the dorsal motor vagal nucleus (DMX) neurons projecting to the porcine stomach prepyloric area in the physiological state and after stomach partial resection. J. Mol. Neurosci..

[26] Huizinga J.D., Martz S., Gil V., Wang X.Y., Jimenez M., Parsons S. (2011). Two independent networks of interstitial cells of cajal work cooperatively with the enteric nervous system to create colonic motor patterns. Front. Neurosci..

[27] Timmermans J.P., Hens J., Adriaensen D. (2001). Outer submucous plexus: an intrinsic nerve network involved in both secretory and motility processes in the intestine of large mammals and humans. Anat. Rec..

[28] Schepp W., Prinz C., Tatge C., Håkanson R., Schusdziarra V., Classen M. (1990). Galanin inhibits gastrin release from isolated rat gastric G-cells. Am. J. Physiol..

[29] Delvaux M., Botella A., Fioramonti J., Frexinos J., Bueno L. (1991). Galanin induces contraction of isolated cells from circular muscle layer of pig ileum. Regul. Pept..

[30] Sternini C., Anselmi L., Guerrini S., Cervio E., Pham T., Balestra B., Vicini R., Baiardi P., D’agostino G.L., Tonini M. (2004). Role of galanin receptor 1 in peristaltic activity in the guinea pig ileum. Neuroscience.

[31] Guerrini S., Raybould H.E., Anselmi L., Agazzi A., Cervio E., Reeve J.R., Tonini M., Sternini C. (2004). Role of galanin receptor 1 in gastric motility in rat. Neurogastroenterol. Motil..

[32] Allescher H.D., Daniel E.E., Dent J., Fox J.E. (1989). Inhibitory function of VIP-PHI and galanin in canine pylorus. Am. J. Physiol..

[33] Bauer F.E., Zintel A., Kenny M.J., Calder D., Ghatei MABloom S.R. (1989). Inhibitory effect of galanin on postprandial gastrointestinal motility and gut hormone release in humans. Gastroenterology.

[34] Botella A., Delvaux M., Bueno L., Frexinos J. (1992). Intracellular pathways triggered by galanin to induce contraction of pig ileum smooth muscle cells. J. Physiol..

[35] Makowska K., Rytel L., Lech P., Osowski A., Kruminis-Kaszkiel E., Gonkowski S. (2018). Cocaine- and amphetamine-regulated transcript (CART) peptide in the enteric nervous system of the porcine esophagus. C. R. Biol..

[36] Mirabella N., Lamanna C., Assisi L., Botte V., Cecio A. (2000). The relationships of nicotinamide adenine dinucleotide phosphate-d to nitric oxide synthase, vasoactive intestinal polypeptide, galanin and pituitary adenylate activating polypeptide in pigeon gut neurons. Neurosci. Lett..

[37] Biancani P., Walsh J.H., Behar J. (1984). Vasoactive intestinal polypeptide. A neurotransmitter for lower esophageal sphincter relaxation. J. Clin. Investig..

[38] Wiley J.W., O’Dorisio T.M., Owyang C. (1988). Vasoactive intestinal polypeptide mediates cholecystokinin-induced relaxation of the sphincter of Oddi. J. Clin. Investig..

[39] Nishio H., Hayashi Y., Terashima S., Takeuchi K. (2006). Role of endogenous nitric oxide in mucosal defense of inflamed rat stomach following iodoacetamide treatment. Life Sci..

[40] Ekblad E. (2006). CART in the enteric nervous system. Peptides.

[41] Kozłowska A., Godlewski J., Majewski M. (2018). Distribution Patterns of Cocaine- and Amphetamine-Regulated Transcript- and/or Galanin-Containing Neurons and Nerve Fibers Located in the Human Stomach Wall Affected by Tumor. Int. J. Mol. Sci..

[42] Vasina V., Barbara G., Talamonti L., Stanghellini V., Corinaldesi R., Tonini M., De Ponti F., De Giorgio R. (2006). Enteric neuroplasticity evoked by inflammation. Auton. Neurosci..

[43] Shipp A., Lawrence G., Gentry R., McDonald T., Bartow H., Bounds J., Macdonald N., Clewell H., Allen B., Van Landingham C. (2006). Acrylamide: review of toxicity data and dose-response analyses for cancer and noncancer effects. Crit. Rev. Toxicol..

[44] Lo Pachin R.M. (2010). The changing view of acrylamide neurotoxicity. Toxicol. Vitro.

[45] Ewaleifoh O., Trinh M., Griffin J.W., Nguyen T. (2012). A novel system to accelerate the progression of nerve degeneration in transgenic mouse models of neuropathies. Exp. Neurol..

[46] Palus K., Bulc M., Całka J. (2018). Changes in VIP-, SP- and CGRP- like immunoreactivity in intramural neurons within the pig stomach following supplementation with low and high doses of acrylamide. Neurotoxicology.

[47] Palus K., Makowska K., Całka J. (2018). Acrylamide-induced alterations in the cocaine- and amphetamine-regulated peptide transcript (CART)-like immunoreactivity within the enteric nervous system of the porcine small intestines. Ann. Anat..

[48] Mahoney S.A., Hosking R., Farrant S., Holmes F.E., Jacoby A.S., Shine J., Iismaa T.P., Scott M.K., Schmidt R., Wynick D. (2003). The second galanin receptor GalR2 plays a key role in neurite outgrowth from adult sensory neurons. J. Neurosci..

[49] Lourenssen S., Miller K.G., Blennerhassett M.G. (2009). Discrete responses of myenteric neurons to structural and functional damage by neurotoxins in vitro. Am. J. Physiol. Gastrointest. Liver Physiol..

[50] Santhanasabapathy R., Vasudevan S., Anupriya K., Pabitha R., Sudhandiran G. (2015). Farnesol quells oxidative stress, reactive gliosis and inflammation during acrylamide-induced neurotoxicity: Behavioral and biochemical evidence. Neuroscience.

[51] Matkowskyj K., Royan S.V., Blunier A., Hecht G., Rao M., Benya R.V. (2009). Age-dependent differences in galanin-dependent colonic fluid secretion after infection with Salmonella typhimurium. Gut.

[52] Dallos A., Kiss M., Polyánka H., Dobozy A., Kemény L., Husz S. (2006). Galanin receptor expression in cultured human keratinocytes and in normal human skin. J. Peripher. Nerv. Syst..

[53] Delgado M., Pozo D., Ganea D. (2004). The significance of vasoactive intestinal peptide in immunomodulation. Pharm. Rev..

[54] Ganea D., Hooper K.M., Kong W. (2015). The neuropeptide vasoactive intestinal peptide: direct effects on immune cells and involvement in inflammatory and autoimmune diseases. Acta Physiol..

[55] Arciszewski M.B., Ekblad E. (2005). Effects of vasoactive intestinal peptide and galanin on survival of cultured porcine myenteric neurons. Regul. Pept..

[56] Lin Z., Sandgren K., Ekblad E. (2004). Increased expression of nitric oxide synthase in cultured neurons from adult rat colonic submucous ganglia. Auton. Neurosci..

[57] Yunker A.M., Galligan J.J. (1998). Extrinsic denervation increases myenteric nitric oxide synthase-containing neurons and inhibitory neuromuscular transmission in guinea pig. J. Auton. Nerv. Syst..

[58] Rychlik A., Gonkowski S., Nowicki M., Calka J. (2017). Inflammatory bowel disease affects density of nitrergic nerve fibers in the mucosal layer of the canine gastrointestinal tract. Can. J. Vet. Res..

[59] Gonkowski S., Burlinski P., Szwajca P., Całka J. (2012). Changes in cocaine- and amphetamine-regulated transcript- like immunoreactive (CART-LI) nerve structures of the porcine descending colon during proliferative enteropathy. Bull. Vet. Inst. Pulawy.

[60] Kasacka I., Piotrowska Z. (2012). Evaluation of density and distribution of CART-immunoreactive structures in gastrointestinal tract of hypertensive rats. Biofactors..

[61] Palus K., Bulc M., Całka J. (2017). Changes in Somatostatin-Like Immunoreactivity in the Sympathetic Neurons Projecting to the Prepyloric Area of the Porcine Stomach Induced by Selected Pathological Conditions. BioMed Res. Int..

